# The Relationship Between Chest Wall Muscle Thickness, Pulmonary Function, and Prognostic Markers in Idiopathic Pulmonary Fibrosis

**DOI:** 10.3390/medicina61071181

**Published:** 2025-06-29

**Authors:** Pelin Pınar Deniz, Sevgül Köse, İsmail Hanta, Pelin Duru Çetinkaya, Merisa Sinem Arslan, Erolcan Datlı

**Affiliations:** 1 Department of Respiratory Disease, Cukurova University Faculty of Medicine, Yüregir, Adana 01250, Turkey; ihanta@cu.edu.tr (İ.H.); pelindurucetinkaya@hotmail.com (P.D.Ç.); merisa.sinem@hotmail.com (M.S.A.); 2 Department of Radiology, Cukurova University Faculty of Medicine, Yüregir, Adana 01250, Turkey; sevgulkarakose@gmail.com (S.K.); erocandatli@gmail.com (E.D.)

**Keywords:** chest wall muscle mass, idiopathic pulmonary fibrosis, prognosis

## Abstract

*Background and Objectives*: Idiopathic pulmonary fibrosis (IPF) is a specific form of chronic, progressive interstitial lung disease with an unknown etiology. It is often accompanied by skeletal muscle mass loss. Chest wall muscles play a crucial role in respiratory movements and form part of the skeletal muscles. The aim of this study is to evaluate the relationship between chest wall muscle thickness and pulmonary function test (PFT) results, as well as other prognostic markers, in patients with IPF. *Materials and Methods*: A retrospective analysis was conducted on 108 patients diagnosed with IPF and 53 control subjects. Chest wall muscle thickness was measured on thoracic computed tomography (CT) images at specific anatomical levels. PFT parameters, the Gender-Age-Physiology (GAP) index, number of acute exacerbations, and mortality data were evaluated in relation to muscle thickness. *Results*: IPF patients had significantly reduced thickness in the bilateral external scapular muscles at both the aortic and pulmonary trunk levels compared to controls. Bilateral pectoral muscle thickness at the aortic level was positively correlated with forced vital capacity (FVC) and negatively correlated with the number of exacerbations. Internal scapular muscle thickness at the aortic level showed a significant positive correlation with diffusion capacity of the lung for carbon monoxide (DLCO) and a negative correlation with both GAP scores and exacerbation frequency. External scapular muscle thickness at the pulmonary trunk level was positively associated with PFT parameters and inversely correlated with the GAP index, exacerbations, and mortality. *Conclusions*: In patients with IPF, the bilateral external scapular muscle thickness at the aortic and pulmonary trunk levels was significantly reduced compared to controls. Significant associations were found between some chest wall muscle thicknesses and the GAP index, pulmonary function, acute exacerbations, and mortality, underscoring the prognostic value of baseline muscle measurements. Measurement of chest wall muscle thickness using routine thoracic CT scans may offer additional prognostic value in IPF. Incorporating this parameter into clinical evaluation may help identify patients who could benefit from supportive interventions, such as nutritional therapy or pulmonary rehabilitation.

## 1. Introduction

Idiopathic pulmonary fibrosis (IPF) is defined as a chronic, progressive fibrotic interstitial pneumonia of unknown etiology, primarily observed in elderly adults [1]. Although the etiology remains unknown, various risk factors such as advanced age, male gender, environmental exposure, and smoking have been identified. In addition, there are studies supporting the potential etiological role of gastroesophageal reflux in pulmonary fibrosis, as well as studies emphasizing the importance of genetic evaluation in the diagnosis of familial pulmonary fibrosis [2,3].

While the average survival time post-diagnosis is reported as 2–5 years, the clinical course is unpredictable. Numerous factors contribute to the worsening clinical course, with acute exacerbations being one of the most significant [4]. The mortality rate from IPF is increasing across Europe, with more than 17,000 deaths recorded annually due to IPF [5]. Generally, advanced age, male gender, worsening dyspnea, and greater lung function impairment are associated with a poorer prognosis [6]. In studies related to interstitial lung disease (ILD), sarcopenia was reported in 32.1% of Japanese patients with ILD. Previous studies evaluating muscle mass in IPF patients have examined parameters such as appendicular skeletal muscle index and the cross-sectional area of erector spinae muscles [7]. These studies have demonstrated that low skeletal muscle mass is associated with reduced quality of life and worse prognosis.

The measurement of skeletal muscle cross-sectional areas on single-slice axial computed tomography (CT) scans offers an alternative method for evaluating regional muscle mass [8]. In patients with advanced lung disease, correlations have been demonstrated between muscle CSA measured at a single thoracic level on chest CT and total muscle volume [9]. Based on these findings, we hypothesized that muscle thickness measurements obtained from routine thoracic CT scans in patients with IPF may reflect overall muscle mass. Furthermore, we proposed that chest wall muscle mass may be reduced in IPF patients and that this reduction could be associated with pulmonary function test (PFT) parameters, which play an important role in monitoring the disease and determining prognosis, as well as other prognostic markers. To enable the measurement of chest wall muscle mass in routine clinical practice without requiring complex programs, we selected chest wall muscle thickness as an indicator of chest wall muscle mass.

## 2. Materials and Methods

**Figure 1:**
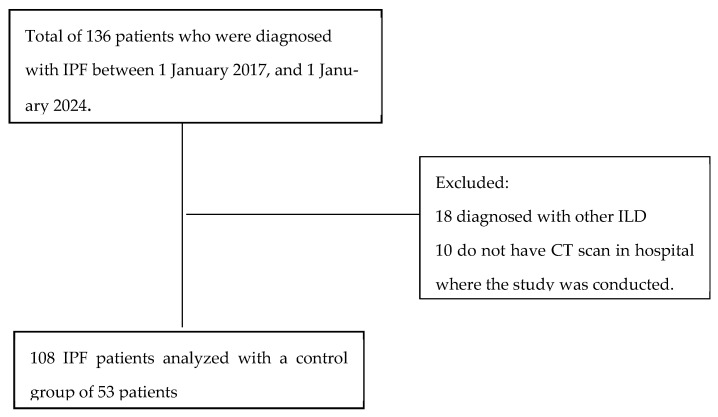
Flow diagram of subjects in this study. Abbreviations; IPF, idiopathic pulmonary fibrosis; ILD, interstitial lung disease; CT, computed tomography.

In this single-center retrospective cross-sectional study, data were examined for 136 patients diagnosed and followed up at our hospital between 1 January 2017 and 1 January 2024. The study included patients diagnosed with IPF in accordance with the official ATS/ERS/JRS/ALAT statement. Figure 1 displays the flow diagram that illustrates the process of selecting the subjects for this study. Eighteen patients were excluded from the study following reevaluation due to indeterminate usual interstitial pneumonia patterns on high-resolution chest computed tomography (HRCT) without histopathological confirmation or radiological findings suggestive of an alternative diagnosis, and 10 patients were excluded due to unavailable HRCT images. The final analysis was conducted with 108 IPF patients and a comparison group of 53 patients. The comparison group consisted of age- and gender-matched individuals with chest CT images taken for screening or nodule follow-up. Additional comorbidities such as a history of cancer, uncontrolled thyroid disease, and muscle disorders were set as exclusion criteria due to their potential impact on muscle mass. Furthermore, patients with chronic respiratory or neurological diseases were excluded from the control group. Comorbidities of both the control group and IPF patients are presented in Table 1.

**Figure 2:**
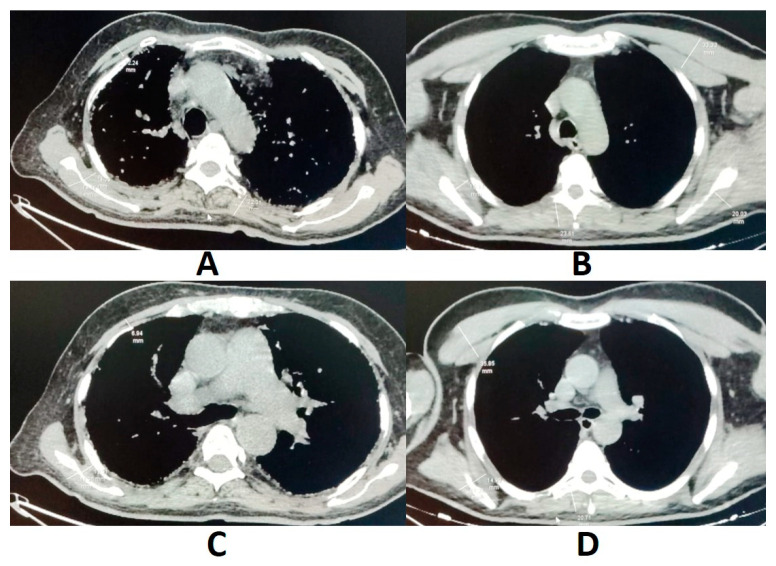
Representative CT slices showing chest wall muscle thickness measurements in IPF patients and controls. Muscle thickness was measured on axial CT images. Arrows indicate measurement points: (**A**,**B**) at the level of the aortic arch; (**C**,**D**) at the level of the pulmonary trunk. Abbreviations; IPF, idiopathic pulmonary fibrosis; CT, computed tomography.

High-resolution chest CT scans obtained from all patients in the supine position without the administration of iodinated contrast were evaluated. Muscle thickness was measured from CT slices using a method similar to that used by Xiaoyan and others [10]. The thickness of the chest wall was measured by radiologists in three sections: at the aortic arch window, at the level of the pulmonary trunk, and at the level of the 10th thoracic vertebra. The thickness of the anterior chest wall muscles, including the major and minor pectoral muscles, the thickness of the internal and external scapular muscles on the posterior chest wall, and the thickness of the erector spinae muscles were measured on both sides of the midclavicular line (Figure 2). Muscle tissues were differentiated from adipose and surrounding tissues by radiologists using Hounsfield unit thresholds, and muscle thickness was manually measured at the predefined anatomical levels.

Pulmonary function test results and diffusion capacity for carbon monoxide (DLco) measurements, performed for all patients using a spirometer (Jeager, Germany) in accordance with ATS/ERS criteria, were obtained from medical records [11]. The Gender-Age-Physiology (GAP) index was calculated using gender, age, percent predicted forced vital capacity (FVC), and percent predicted DLco, based on the formula proposed by Ley et al. [12].

Gender-Age-Physiology index at diagnosis, PFT values, body mass index (BMI), hospitalization history, acute exacerbation count during follow-up, and mortality data were recorded. Participants with low-quality CT images, implants, or pacemakers that affected imaging quality were excluded from the study. Demographic and clinical data were retrospectively obtained from patients’ medical records.

### 2.1. Statistical Analysis

Continuous variables were expressed as the mean ± standard deviation, and categorical data were expressed as frequency and percentage. The Kolmogorov–Smirnov goodness-of-fit test was used to analyze normality for the intergroup analysis of continuous variables. As data followed a normal distribution, Student’s *t*-test was used for intergroup comparisons. A chi-square test was used for categorical variable comparisons. The Pearson correlation test was used to assess the linear relationship between variables. Statistical analyses were performed using IBM SPSS version 27.0 (IBM Corporation, Armonk, NY, USA), and statistical significance was set at *p* < 0.05.

### 2.2. Ethics Approval and Consent to Participate

The study protocol was approved by the Ethical Committee of the Cukurova University School of Medicine (146/30) and was conducted in accordance with the approved guidelines. The Ethical Committee of Cukurova University School of Medicine waived the need for patient approval and/or informed consent due to the retrospective nature of the study.

## 3. Results

The study included 108 IPF patients and a control group of 53 individuals. There was no statistically significant difference in age, gender, or presence of chronic illness between the IPF and control groups (*p* > 0.05) (Table 1).

In IPF patients, the thickness of the right and left exterior scapular muscles at the aortic arch level was significantly lower compared to the control group (21.84 ± 5.75 and 21.83 ± 5.78 versus 27.35 ± 6.58 and 27.33 ± 6.59, respectively; *p* < 0.001 for both). Similarly, at the pulmonary trunk region, right and left exterior scapular muscle thickness in IPF patients was significantly lower than that in the control group (19.56 ± 5.04 and 19.60 ± 5.06 versus 23.39 ± 7.49 and 23.43 ± 7.46, respectively; *p* = 0.001 for both) (Table 2).

We looked at the relationship between the thickness of the chest wall muscles in the aortic region and pulmonary function tests, the GAP index, the number of acute exacerbations, and death in IPF patients. The thickness of the right and left aortic pectoral muscles had a positive and statistically significant relationship with FVC (L) (r = 0.229, *p* = 0.031, and r = 0.230, *p* = 0.030, respectively) and a negative and statistically significant relationship with the number of acute exacerbations (r = −0.267, *p* = 0.006, and r = −0.273, *p* = 0.005, respectively). The thickness of the right and left aortic inner scapular muscles demonstrated a positive and statistically significant correlation with DLCO (%) (r = 0.252, *p* = 0.012, and r = 0.254, *p* = 0.011, respectively) and a negative and statistically significant correlation with GAP index and the number of acute exacerbations (for GAP index: r = −0.219, *p* = 0.029, and r = −0.210, *p* = 0.036; for acute exacerbations: r = −0.242, *p* = 0.013, and r = −0.247, *p* = 0.011). The thickness of the right aortic exterior scapular muscle was positively and significantly correlated with DLCO (%) (r = 0.238, *p* = 0.017) and negatively and significantly correlated with GAP index and the number of acute exacerbations (r = −0.318, *p* = 0.001, and r = −0.268, *p* = 0.006, respectively). Similarly, the thickness of the left aortic exterior scapular muscle was positively and significantly correlated with DLCO (%) (r = 0.239, *p* = 0.017) and negatively and significantly correlated with GAP stage, the number of acute exacerbations, and mortality (r = −0.317, *p* = 0.001, r = −0.263, *p* = 0.007, and r = −0.190, *p* = 0.049, respectively). The thickness of bilateral aortic erector spinae muscles showed a negative and statistically significant correlation with GAP stage and the number of acute exacerbations (for the right side: r = −0.231, *p* = 0.021, and r = −0.207, *p* = 0.034; for the left side: r = −0.231, *p* = 0.021, and r = −0.206, *p* = 0.035).

In the pulmonary trunk region, the thickness of the right exterior scapular muscle was positively and significantly correlated with FVC (L) and DLCO (%) (r = 0.254, *p* = 0.016, and r = 0.354, *p* < 0.001, respectively) and negatively and significantly correlated with GAP stage, the number of acute exacerbations, and mortality (r = −0.320, *p* = 0.001, r = −0.374, *p* < 0.001, and r = −0.261, *p* = 0.006, respectively). The thickness of the left exterior scapular muscle was positively and significantly correlated with FVC (L) and DLCO (%) (r = 0.258, *p* = 0.015, and r = 0.356, *p* < 0.001, respectively) and negatively and significantly correlated with GAP stage, the number of acute exacerbations, and mortality (r = −0.316, *p* = 0.001, r = −0.375, *p* < 0.001, and r = −0.258, *p* = 0.007, respectively). The thickness of the right erector spinae muscle showed a positive and statistically significant correlation with FVC (L) (r = 0.278, *p* = 0.008) and a negative and statistically significant correlation with GAP stage and mortality (r = −0.350, *p* < 0.001, and r = −0.210, *p* = 0.029, respectively). Similarly, the thickness of the left erector spinae muscle demonstrated a positive and statistically significant correlation with FVC (L) (r = 0.276, *p* = 0.009) and a negative and statistically significant correlation with GAP stage and mortality (r = −0.348, *p* < 0.001, and r = −0.209, *p* = 0.030, respectively) (Table 3).

When evaluating the impact of erector spinae muscle thickness at the level of the 10th thoracic vertebra on FVC, DLCO, GAP index, acute exacerbations, and mortality, no statistically significant results were found.

## 4. Discussion

In patients with idiopathic IPF, the thickness of the bilateral external scapular muscles at the aortic and pulmonary trunk levels was significantly thinner compared to the control group. A notable relationship was observed between certain chest wall muscle thicknesses and the GAP index, PFT parameters, frequency of acute exacerbations, and mortality. This finding highlights the importance of initial chest wall muscle measurements in IPF patients. Chest CT is a routinely performed and practical diagnostic tool at the time of diagnosis for all patients with IPF. We believe that the measurement of chest wall muscle thickness from CT images can be easily adapted to clinical practice without adding an extra financial burden on the healthcare system.

In our study, the thickness of the right and left external scapular muscles at the level of the aortic arch and pulmonary trunk was found to be significantly lower in IPF patients compared to the control group. Although muscle thickness appeared to be reduced in all measured regions in the IPF group—except for the erector spinae muscle—only the right and left external scapular muscle thicknesses reached statistical significance. This may be due to the limited sample size. Our study observed that chest muscle thickness in IPF patients was lower than that in the control group, suggesting a suspicion of sarcopenia in these patients. Sarcopenia is defined as an age-related syndrome characterized by a progressive and generalized loss of skeletal muscle mass and strength. The presence of sarcopenia is associated with physical disability, reduced quality of life, and increased risk of mortality [13]. The decrease in skeletal muscle mass is thought to be triggered by inflammation, inactivity, malnutrition, and increased energy expenditure. In lung diseases, factors such as impaired lung function, exercise-induced hypoxemia, and poor nutritional status may also contribute to inactivity, leading to sarcopenia. Furthermore, sarcopenia affects respiratory function, motor skills, swallowing profiles, and immune responses [14,15]. Its prevalence in chronic obstructive pulmonary disease (COPD) has been reported to range from 15.5% to 21.6% [16]. In ILD, sarcopenia has been identified in 32.1% of Japanese patients [17]. In a Japanese population-based study, the prevalence of sarcopenia among older adults was reported as 11.5% in men and 16.7% in women [18]. These rates suggest that sarcopenia may be more prevalent in patients with IPF compared to the general population. Reduced skeletal muscle mass in these patients has been associated with a lower quality of life and a worse prognosis. In the study by Fujita et al., sarcopenia was identified in 39.3% of patients with IPF. Sarcopenia in IPF patients was found to be associated with age, FVC, and GAP index [7]. Additionally, when comparing sarcopenic and non-sarcopenic IPF patients, significant differences were observed in quality of life, dyspnea, and depression scores, with sarcopenic patients showing worse outcomes. Depression, which is notably prevalent in sarcopenic patients, was found to significantly impact the quality of life in those with IPF [19,20]. We believe that due to the impact of sarcopenia on IPF patients, it is important to detect it at the time of diagnosis.

In patients with advanced lung disease, correlations have been reported between the cross-sectional area (CSA) of thoracic muscles measured at a single level on chest CT and total muscle mass [9]. Based on this, Rozenberg et al. observed that the CSA of the pectoralis, intercostal, paraspinal, serratus, and latissimus muscles at the level of the carina—corresponding to the fourth thoracic vertebra—was associated with clinical indicators of frailty in lung transplant patients, including six-minute walk distance, biceps and quadriceps training volumes, and length of hospital stay [21]. Fuseya et al. reported that the CSA of the erector spinae muscle at the level of the 12th thoracic vertebra (ESMCSA) was associated with severe airflow limitation, respiratory symptoms, emphysema severity, and mortality in patients with COPD [9]. In a study by Moon et al., the CSA of the pectoralis, paraspinal, serratus, and latissimus muscles at the level of the fourth thoracic vertebra and the erector spinae muscle at the 12th vertebral level were analyzed in patients with IPF. Their findings demonstrated that low thoracic skeletal muscle mass, normalized by body height and measured at the level of the fourth vertebra on chest CT, was a strong risk factor for all-cause mortality in patients with IPF [22].

Studies evaluating muscle CSA in respiratory diseases commonly focus on the erector spinae muscle at the level of the 12th thoracic vertebra and the pectoralis muscle above the aortic arch [9,22,23,24]. These muscles are often selected because they can be assessed using routine thoracic CT scans without the need for additional radiation exposure. Given the distinct functional roles of these muscle groups, changes in the pectoral and scapular muscles, which play a more active role in respiratory function, are more likely to reflect clinical and physiological impairment. In contrast, the erector spinae muscle, primarily responsible for trunk extension and postural support, may reflect general physical functioning rather than respiratory-specific decline.

In our study, muscle measurements were obtained only at baseline, and no follow-up imaging data were available. As a result, it is not possible to determine whether greater muscle loss occurred as a consequence of disease progression or whether a greater degree of muscle wasting over time might have contributed to a more rapid progression of the disease. Clarifying this relationship will require prospective, longitudinal studies that include serial imaging assessments.

The method we used to measure chest wall muscle thickness was similar to that of Xiaoyan et al., who evaluated chest wall muscle thickness in patients with bronchiectasis. However, while Xiaoyan et al. performed measurements above the aortic arch, we chose to measure at the pulmonary trunk level in order to minimize the impact of adipose tissue between the muscles in this region on the measurements. Furthermore, unlike Xiaoyan et al., we selected the T10 level as our third measurement site instead of T12, considering that T12 might not always be included in routine CT slices and to facilitate easier measurements [10].

In our study, muscle thickness measurements were performed manually. While our measurements were obtained from anatomically predefined regions, the manual nature of the assessment may not be as sensitive or accurate as imaging-based techniques specifically designed to assess sarcopenia. This might also explain why some measurement sites showed stronger associations than others.

In our study, certain chest wall muscle thicknesses measured at the aortic and pulmonary trunk levels were found to be associated with FVC (L), DLCO (%), and the GAP index. Additionally, some muscle thickness measurements were related to the number of acute exacerbations and mortality, although these correlations were weak. Acute exacerbation of IPF is defined as an acute, clinically significant respiratory deterioration characterized by new widespread alveolar abnormalities. Patient-related factors associated with a higher risk of acute exacerbation include low FVC, reduced DLCO, decreased six-minute walk distance, pulmonary hypertension, poor baseline oxygenation, increased dyspnea, and a decline in FVC during follow-up [25]. Following an acute exacerbation, the median survival is approximately 3 to 4 months, and in-hospital mortality rates can reach up to 50% [26]. This highlights the importance of early prediction of exacerbations and the implementation of preventive strategies, particularly in high-risk patient groups. Although no large-scale studies have specifically addressed the direct impact of sarcopenia on acute exacerbations of IPF, a study by Ito et al. examined the relationship between skeletal muscle mass and 90-day mortality during acute exacerbations. The study demonstrated that patients presenting with lower ESMCSA during AE-IPF had significantly poorer survival outcomes. Sarcopenia has been suggested to be a potential risk factor for acute exacerbations. Therefore, identifying sarcopenia in clinical practice could provide an opportunity to stratify risk and guide targeted interventions in patients with IPF.

In IPF patients, the literature data regarding the pectoral muscles and erector spinae muscles are controversial. In the study by Molgat-Seon et al., the pectoral muscle area (PMA) was found to be associated with FVC (%), DLCO, SpO2, and dyspnea in patients with ILD, even after adjusting for variables such as age, sex, height, body mass, and prednisone use. Additionally, the annual decline in PMA was associated with all-cause mortality, suggesting that a reduced PMA may reflect ILD severity. In this study, a specialized program was used to calculate PMA, with the area measured through these calculations [27]. Ebihara et al. evaluated the appendicular skeletal muscle mass index (ASMI) using dual-energy X-ray absorptiometry (DXA), finding it to have a stronger correlation with various outcome measures in IPF compared to the cross-sectional area of individual muscles. Among muscle groups, the cross-sectional area of the pectoralis major was more strongly correlated with physical performance than the erector spinae muscles [28]. In the study by Avano et al., it was observed that a low ESMCSA is a risk factor for all-cause mortality in IPF patients, independent of established prognostic factors such as age, acute exacerbations, and impaired lung function [24]. Durdu et al. demonstrated that pectoralis muscle strength in IPF patients was significantly lower than in healthy controls. A significant correlation was found between pectoralis muscle strength and measures such as maximal inspiratory and expiratory pressure, FEV1 (%), FVC (%), and DLCO. This study assessed pectoral muscle strength using a handheld dynamometer [29].

Imaging methods commonly used to evaluate sarcopenia include magnetic resonance imaging, CT, DXA, and ultrasonography. Due to its high resolution, CT enables accurate measurement of muscle mass. Furthermore, muscle density can also be assessed using CT, providing valuable information about muscle quality. Specialized software programs are available to measure skeletal muscle mass in each CT slice [30]. However, all these methods involve additional radiation exposure, high costs, or significant time investment. Chest CT, on the other hand, is used in the diagnosis and follow-up of IPF patients and is widely available in most hospitals, making it a convenient tool. Therefore, assessing muscle thickness from chest CT images does not incur additional costs or workload at the time of diagnosis or during follow-up.

The ATS/ERS/JRS/ALAT clinical practice guideline recommends two pharmacologic antifibrotic agents for IPF: nintedanib and pirfenidone. Both drugs are associated with adverse effects that may impair appetite and gastrointestinal absorption, potentially leading to weight loss and malnutrition. Evidence shows that more than 5% weight loss occurs in approximately 20% of IPF patients, even in the absence of antifibrotic therapy [31]. In a study by Perelas et al., both antifibrotic drug selection and disease severity were found to be predictors of weight loss. A clinically significant weight loss after one year of continuous treatment was observed in a higher proportion of patients receiving nintedanib (61%) compared to those receiving pirfenidone (30%) [32]. Nintedanib has been particularly associated with gastrointestinal adverse events, especially diarrhea [33]. In real-world studies of pirfenidone, anorexia and gastrointestinal discomfort were the most common reasons for treatment discontinuation [34]. Considering the gastrointestinal and weight loss effects of antifibrotic therapies, we believe that close monitoring of adverse effects, implementation of nutritional counseling, and prevention of weight loss are essential components of follow-up care in IPF patients who are already at increased risk for sarcopenia.

This study had several limitations. First, it was a retrospective, single-center study. The focus on a single ethnic population also limits the generalizability of the findings and may introduce bias, as BMI and the prevalence of sarcopenia vary across ethnic groups. Multicenter studies involving diverse populations would help validate and generalize the results. Second, the radiologists were not blinded to the patients’ diagnoses, as parenchymal pathologies were visible during the measurements, which may have introduced bias. However, the 28 excluded patients—due to diagnostic inconsistencies or lack of adequate CT images—were removed prior to radiologic evaluation, reducing the risk of selection bias. Third, the control group included only radiological data, and PFT and BMI data could not be compared with the IPF group. Although the control and IPF groups were similar in terms of age, gender, and comorbidities, BMI data for the control group were unavailable for analysis.

## 5. Conclusions

In conclusion, our study demonstrated that chest muscle thicknesses were reduced in IPF patients compared to the control group. Some chest muscle thickness measurements in IPF patients were found to have a low level of correlation with FVC, DLCO, GAP index, acute exacerbation frequency, and mortality data. Utilizing chest CT, which is routinely part of the diagnostic algorithm for IPF, to evaluate chest muscle thickness may offer additional prognostic insights. By measuring muscle thickness at diagnosis, it may be possible to identify patients who could benefit from interventions such as nutritional support and pulmonary rehabilitation, potentially contributing to an improved prognosis. Moreover, incorporating these measurements into both the diagnostic and follow-up processes may provide further benefits in the management of IPF patients.

## Figures and Tables

**Table 1:** Comparison of socio-demographic and clinical characteristics of the groups.

	Control (*n* = 53)	IPF Patients (*n* = 108)	*p*
Age (years) (mean ± SD)	63.66 ± 8.68	66.43 ± 8.46	0.054 *
Gender (*n*, %)			
Male	46 (86.8%)	94 (87.0%)	0.965 **
Female	7 (13.2%)	14 (13.0%)	
Presence of chronic illness (n, %)			
Coronary Artery Disease	18 (34.0%)	50 (46.3%)	0.137 **
Hypertension	27 (50.9%)	43 (39.8%)	0.181 **
Diabetes Mellitus	17 (32.1%)	32 (29.6%)	0.751 **

* Student’s *t* test; ** chi-square test. Abbreviations: SD: standard deviation; IPF: idiopathic pulmonary fibrosis.

**Table 2:** Comparison of chest wall muscle thickness values between IPF and control group.

	Control (Mean ± SD) (*n* = 53)	IPF (Mean ± SD) (*n* = 108)	*p*
Aortic arch window (mm)			
Aortic pectoral (right)	20.26 ± 6.16	19.49 ± 5.52	0.423 *
Aortic pectoral (left)	20.26 ± 6.14	19.46 ± 5.58	0.409 *
Aortic interior scapular (right)	19.11 ± 6.33	17.89 ± 5.20	0.198*
Aortic interior scapular (left)	19.13 ± 6.32	17.88 ± 5.21	0.188 *
Aortic exterior scapular (right)	27.35 ± 6.58	**21.84 ± 5.75**	<0.001 *
Aortic exterior scapular (left)	27.33 ± 6.59	**21.83 ± 5.78**	<0.001 *
Erector spinae muscle (right)	27.33 ± 4.65	27.36 ± 5.36	0.980 *
Erector spinae muscle (left)	27.33 ± 4.65	27.35 ± 5.37	0.989 *
**Pulmonary trunk**			
Pectoral (right)	12.77 ± 7.29	11.43 ± 4.03	0.217 *
Pectoral (left)	12.73 ± 7.27	11.39 ± 4.07	0.217 *
Interior scapular (right)	15.03 ± 5.50	13.82 ± 4.59	0.143 *
Interior scapular (left)	15.07 ± 5.50	13.84 ± 4.62	0.138 *
Exterior scapular (right)	23.39 ± 7.49	**19.56 ± 5.04**	0.001 *
Exterior scapular (left)	23.43 ± 7.46	**19.60 ± 5.06**	0.001 *
Erector spinae muscle (right)	25.50 ± 4.94	24.67 ± 5.43	0.348 *
Erector spinae muscle (left)	25.47 ± 4.94	24.66 ± 5.42	0.364 *
**10th thoracic vertebra level**			
Erector spinae muscle (right)	22.22 ± 4.15	22.57 ± 4.58	0.642 *
Erector spinae muscle (left)	22.22 ± 4.15	22.58 ± 4.57	0.633 *

* Student’s *t*-test. Abbreviations: SD: standard deviation; IPF: idiopathic pulmonary fibrosis. Bold characters are used to stressed significant data.

**Table 3:** Correlation between chest wall muscle thickness and pulmonary function tests, GAP index, acute exacerbation count, and mortality in IPF patients.

	Correlation Index (r)	*p*		Correlation Index (r)	*p*
**Aortic Pectoral (Right)**			**Pulmonary Trunk Pectoral (Right)**		
FVC (%)	0.044	0.661 *	FVC (%)	−0.055	0.583 *
FVC (L)	0.229	0.031 *	FVC (L)	−0.013	0.901 *
DLCO (%)	0.115	0.253 *	DLCO (%)	0.092	0.361 *
GAP Stage	−0.178	0.077 *	GAP Stage	−0.145	0.150 *
Acute exacerbation count	−0.267	0.006 *	Acute exacerbation count	−0.090	0.361 *
Mortality	−0.111	0.255 *	Mortality	0.110	0.259 *
**Aortic pectoral (left)**			**Pulmonary trunk pectoral (left)**		
FVC (%)	0.053	0.599 *	FVC (%)	−0.030	0.764 *
FVC (L)	0.230	0.030 *	FVC (L)	0.010	0.927 *
DLCO (%)	0.122	0.227 *	DLCO (%)	0.108	0.284 *
GAP Stage	−0.175	0.081 *	GAP Stage	−0.149	0.138 *
Acute exacerbation count	−0.273	0.005 *	Acute exacerbation count	−0.105	0.285 *
Mortality	−0.115	0.235 *	Mortality	0.093	0.340 *
**Aortic inner scapular (right)**			**Pulmonary trunk inner** **scapular (right)**		
FVC (%)	0.061	0.544 *	FVC (%)	−0.020	0.843 *
FVC (L)	0.151	0.158 *	FVC (L)	0.094	0.381 *
DLCO (%)	0.252	0.012 *	DLCO (%)	0.166	0.099 *
GAP Stage	−0.219	0.029 *	GAP Stage	−0.163	0.105 *
Acute exacerbation count	−0.242	0.013 *	Acute exacerbation count	−0.118	0.229 *
Mortality	−0.148	0.126 *	Mortality	−0.047	0.627 *
**Aortic inner scapular (left)**			**Pulmonary trunk inner** **scapular (left)**		
FVC (%)	0.058	0.562 *	FVC (%)	−0.015	0.879 *
FVC (L)	0.151	0.157 *	FVC (L)	0.107	0.320 *
DLCO (%)	0.254	0.011 *	DLCO (%)	0.169	0.093 *
GAP Stage	−0.210	0.036 *	GAP Stage	−0.175	0.082 *
Acute exacerbation count	−0.247	0.011 *	Acute exacerbation count	−0.123	0.210 *
Mortality	−0.150	0.121 *	Mortality	−0.058	0.548*
**Aortic outer scapular (right)**			**Pulmonary trunk outer scapular (right)**		
FVC (%)	0.055	0.583 *	FVC (%)	0.120	0.230 *
FVC (L)	0.201	0.059 *	FVC (L)	0.254	0.016 *
DLCO (%)	0.238	0.017 *	DLCO (%)	0.354	<0.001 *
GAP Stage	−0.318	0.001 *	GAP Stage	−0.320	0.001 *
Acute exacerbation count	−0.268	0.006 *	Acute exacerbation count	−0.374	<0.001 *
Mortality	−0.186	0.055 *	Mortality	−0.261	0.006 *
**Aortic outer scapular (left)**			**Pulmonary trunk outer scapular (left)**		
FVC (%)	0.058	0.560 *	FVC (%)	0.138	0.167 *
FVC (L)	0.206	0.053 *	FVC (L)	0.258	0.015 *
DLCO (%)	0.239	0.017 *	DLCO (%)	0.356	<0.001 *
GAP Stage	−0.317	0.001 *	GAP Stage	−0.316	0.001 *
Acute exacerbation count	−0.263	0.007 *	Acute exacerbation count	−0.375	<0.001 *
Mortality	−0.190	0.049 *	Mortality	−0.258	0.007 *
**Aortic** **erector spinae muscle (right)**			**Pulmonary trunk erector spinae muscle (right)**		
FVC (%)	−0.022	0.823 *	FVC (%)	0.083	0.407 *
FVC (L)	0.125	0.241 *	FVC (L)	0.278	0.008 *
DLCO (%)	0.149	0.139 *	DLCO (%)	0.185	0.066 *
GAP Stage	−0.231	0.021 *	GAP Stage	−0.350	<0.001 *
Acute exacerbation count	−0.207	0.034 *	Acute exacerbation count	−0.133	0.178 *
Mortality	−0.147	0.129 *	Mortality	−0.210	0.029 *
**Aortic** **erector spinae muscle (left)**			**Pulmonary trunk erector spinae muscle (left)**		
FVC (%)	−0.029	0.771 *	FVC (%)	0.080	0.423 *
FVC (L)	0.128	0.232 *	FVC (L)	0.276	0.009 *
DLCO (%)	0.144	0.154 *	DLCO (%)	0.181	0.071 *
GAP Stage	−0.231	0.021 *	GAP Stage	−0.348	**<0.001** *
Acute exacerbation count	−0.206	0.035 *	Acute exacerbation count	−0.132	0.181 *
Mortality	−0.149	0.124 *	Mortality	−0.209	**0.030** *

* Pearson correlation analysis. Abbreviations: GAP, Gender-Age-Physiology; IPF, idiopathic pulmonary fibrosis; FVC, forced vital capacity; DLCO, diffusion capacity of the lungs for carbon monoxide. Bold characters are used to stressed significant data.

## Data Availability

The datasets used and analyzed during the current study are available from the corresponding author on reasonable request.

## Abbreviations

The following abbreviations are used in this manuscript:

IPF	Idiopathic pulmonary fibrosis
PFT	Pulmonary function test
CT	Computed tomography
DLCO	Diffusing capacity of carbon monoxide
FEV1	Forced expiratory volume
FV	Forced vital capacity
GAP index	Gender-Age-Physiology Index
HRCT	High-resolution chest computed tomography
ILD	Interstitial lung disease
SD	Standard deviation
CSA	Cross-sectional area
PMA	Pectoral muscle area
ASMI	Appendicular skeletal muscle mass index
DXA	Dual-energy X-ray absorptiometry
ESM_CSA_	Cross-sectional area of the erector spinae muscle
BMI	Body mass index
